# Perimesencephalic Subarachnoid Hemorrhage Bleeding Patterns Are Not Always Benign: Prognostic Impact of an Aneurysmal Pathology

**DOI:** 10.3390/biomedicines13061444

**Published:** 2025-06-12

**Authors:** Emily Hoffmann, Công Dùy Bui, David Ventura, Manfred Musigmann, Alexandra Valls Chavarria, Markus Holling, Vivek S. Yedavalli, Jeremy J. Heit, Christian Paul Stracke, Tobias D. Faizy, Hermann Krähling, Burak Han Akkurt

**Affiliations:** 1 Clinic of Radiology, University of Münster, 48149 Münster, Germany; 2 Department of Nuclear Medicine, University of Münster, 48149 Münster, Germany; 3 Department of Neurosurgery, University of Münster, 48149 Münster, Germany; 4 Russell H. Morgan Department of Radiology and Radiological Sciences, Johns Hopkins University, Baltimore, MD 21287, USA; 5 Department of Neuroradiology, Stanford University, Stanford, CA 94304, USA; 6 Division of Interventional Neuroradiology, University of Münster, 48149 Münster, Germany

**Keywords:** perimesencephalic subarachnoid hemorrhage, aneurysm, outcome, hydrocephalus, vasospasm, delayed cerebral ischemia

## Abstract

**Background/Objectives**: Perimesencephalic subarachnoid hemorrhage (pmSAH) is generally considered to be a benign variant of spontaneous SAH. However, in rare cases, an underlying aneurysm may be present, altering both clinical management and prognosis. The aim of this study was to evaluate the prognostic impact of aneurysmal pathology in patients presenting with perimesencephalic hemorrhage, focusing on the occurrence of complications and functional outcomes. **Methods**: This single-center, retrospective study included 77 patients diagnosed with perimesencephalic hemorrhage between 2012 and 2022. Clinical and radiological data were extracted, including demographics, risk factors, complications (hydrocephalus, vasospasm, and delayed cerebral ischemia (DCI)), and outcome scores (Glasgow Outcome Scale (GOS) and modified Rankin scale (mRS) at discharge). Patients were divided into two groups based on the presence or absence of an aneurysm confirmed through digital subtraction angiography (DSA). **Results**: Of the 77 patients, 7 (9.1%) were found to have an aneurysm. While rates of complications such as hydrocephalus and DCI were higher in the aneurysm group, these differences did not reach statistical significance. However, patients with aneurysms had significantly worse functional outcomes, with higher mRS and lower GOS scores at discharge. Logistic regression confirmed the presence of aneurysms as an independent factor associated with poor outcomes (OR = 21.6; 95% CI: 1.00−467.3; *p* = 0.050), while other variables such as age, sex, and World Federation of Neurosurgical Societies (WFNS) score were not statistically significant. ROC analysis showed moderate discriminative power of aneurysm presence for poor outcomes (AUC = 0.72). **Conclusions**: The presence of an aneurysm, although rare in pmSAH, significantly worsens functional outcomes. These findings highlight the necessity of early and sensitive vascular diagnostics—particularly DSA—to reliably exclude aneurysms. Differentiating between aneurysmal and non-aneurysmal perimesencephalic bleeding is essential not only for clinical decision-making but also for optimizing resource allocation in neurocritical care.

## 1. Introduction

Perimesencephalic subarachnoid hemorrhage (pmSAH) is a distinct form of subarachnoid hemorrhage characterized by the accumulation of blood in the basal cisterns, typically restricted to the area in front of the midbrain or pons, with the potential for extension into the suprasellar cisterns or proximal Sylvian fissures [1]. This condition generally manifests without significant intraventricular or parenchymal involvement [2]. The incidence of pmSAH is estimated to be approximately 0.5 cases per 100,000 person–years, making it a relatively rare entity. It accounts for approximately 5% to 10% of all spontaneous SAH cases [3].

The initial diagnosis of pmSAH is based on non-contrast head CT (ncCT) imaging to discern the distinctive perimesencephalic hemorrhage pattern [1,4,5]. Subsequent vascular imaging is crucial to exclude aneurysmal causes. Digital subtraction angiography (DSA) is widely regarded as the gold standard, but high-resolution computed tomography angiography (CTA) has emerged as an alternative with high sensitivity and specificity [6,7]. Although pmSAH is frequently regarded as a benign entity, up to 7% of cases may harbor a ruptured aneurysm [3], which necessitates immediate intervention [8]. Thus, the characterization of pmSAH as a “benign” variant of SAH is primarily based on its low rebleeding rates and favorable short-term outcomes [5,9,10]. However, this generalization may underestimate the clinical significance of cases in which the bleeding pattern mimics pmSAH, yet the underlying cause is, in fact, aneurysmal. Furthermore, complications such as hydrocephalus, vasospasm, and delayed cerebral ischemia (DCI) can still occur—even in patients without the presence of an aneurysm [2,11,12,13]. Consequently, the clinical course of patients diagnosed with pmSAH may exhibit greater variability than previously postulated.

The extent to which these complications and functional outcomes vary by etiology (aneurysmal vs. non-aneurysmal pmSAH) remains less well understood. Consequently, the presence of a perimesencephalic bleeding pattern should not lead to premature reassurance, since it is imperative to consider the possibility of an underlying aneurysm.

The aim of this study is to assess the frequency of the presence of an aneurysm as the underlying cause of pmSAH. We sought to investigate the impact of an aneurysmal pathology on the occurrence of complications and functional outcomes following perimesencephalic bleeding, determined based on the Glasgow Outcome Scale (GOS) and modified Rankin scale (mRS) at discharge. By identifying prognostic differences between patients with aneurysmal and non-aneurysmal causes of pmSAH, this analysis seeks to support early risk stratification and clinical management, including monitoring strategies and treatment decisions.

## 2. Materials and Methods

### 2.1. Patient Selection and Study Design

This retrospective, single-center study included all consecutive patients presenting with a perimesencephalic bleeding pattern on admission imaging at a tertiary care academic medical center in Germany between 2012 and 2022. Patients eligible for this study were identified using the search mask of the local radiology information system, including the keywords ‘perimesencephalic’ or ‘prepontine’ and ‘subarachnoid hemorrhage’. Patients were included if they were ≥18 years of age, of either sex, had a diagnosis of perimesencephalic bleeding based on ncCT, and if complete clinical data were available from the electronic medical record (ORBIS), including GOS and mRS.

The study was conducted in accordance with the tenets of the Declaration of Helsinki (as revised in 2013), and the study was approved by the local ethics committee (ID: 2023-415-f-S, 11 October 2023). Due to the retrospective nature of the study, the need for informed consent from patients was waived.

Initial imaging consisted of an ncCT scan followed by CTA of the supra-aortic and cerebral vasculature (80 cc of contrast, 4 cc/s flow rate, ULTRAVIST, Bayer Vital GmbH, Leverkusen, Germany) with the patient’s arms lowered. DSA of the cerebral arteries was performed via a transfemoral arterial approach under general anesthesia using a biplane state-of-the-art angiography unit. At our institution, all patients presenting with a perimesencephalic bleeding pattern undergo DSA within 24 h of admission. If the initial results are unclear or inconclusive, a second DSA is performed to ensure diagnostic accuracy and rule out subtle vascular pathologies.

All patients were treated in accordance with the internal guidelines for patients presenting with SAH-like symptoms, including vasospasm prophylaxis with nimodipine.

### 2.2. Data Collection and Analysis

Imaging data, including ncCT, CTA, and DSA, were gathered and reviewed for complications identifiable on each modality, as well as any underlying vascular pathology. In addition, clinical data were collected from the patients’ medical records, including age, sex, cardiovascular risk factors (arterial hypertension, smoking, and extensive alcohol consumption), clinical symptoms (headache, nausea, vomiting, or neck stiffness), complications during the clinical course (hydrocephalus, assessed based on enlarged ventricles on baseline or follow-up imaging; vasospasm, radiologically or clinically confirmed cerebral artery narrowing with delayed perfusion; and DCI, defined as either new clinical symptoms consistent with cerebral ischemia or new ischemia detected on imaging, provided no alternative explanation such as procedural, epileptic or metabolic causes were present) as well as neurological scores (World Federation of Neurosurgical Societies (WFNS) score, Glasgow Outcome Scale (GOS), and modified Ranking scale (mRS), assessed at discharge, respectively).

All imaging data were analyzed independently by two radiologists with four and six years of neuroimaging experience. In cases of initial disagreement, the findings were discussed jointly until consensus was reached. While this approach ensured a consistent interpretation, it did not systematically assess quantitative measures of inter-rater reliability. Neurological assessments were performed by trained neurosurgeons.

### 2.3. Statistical Analysis

Data are presented as the mean ± standard deviation (SD) for normally distributed continuous variables or as the median and interquartile range (IQR) if non-normally distributed; ordinal variables are consistently summarized using medians and IQR. Normality was assessed using Shapiro–Wilk tests. For group comparisons, Mann–Whitney U tests were used for continuous and ordinal variables, and chi-squared or Fisher’s exact tests were used for categorical variables.

To identify independent factors associated with poor functional outcomes (defined as mRS >2 at discharge), a multivariable binary logistic regression model was constructed, including aneurysm status, age, sex, and WFNS score as covariates. In addition, receiver operating characteristic (ROC) analysis was performed to evaluate the discriminative ability of aneurysm presence in predicting poor outcomes. *p*-values  <  0.05 were considered statistically significant. All statistical analyses were performed using GraphPad Prism (version 9.2.0, GraphPad Software Inc., San Diego, CA, USA) and SPSS Statistics version 29 (SPSS Inc., Chicago, IL, USA).

## 3. Results

### 3.1. Study Cohort

From the 183 patients screened for this study, 77 patients with a perimesencephalic bleeding pattern (59.7% male) and a mean age of 55.2 ± 11.9 years were enrolled. The other 106 patients were excluded due to a non-perimesencephalic pattern of hemorrhage, incomplete medical records, or poor quality of diagnostic imaging.

**Figure 1:**
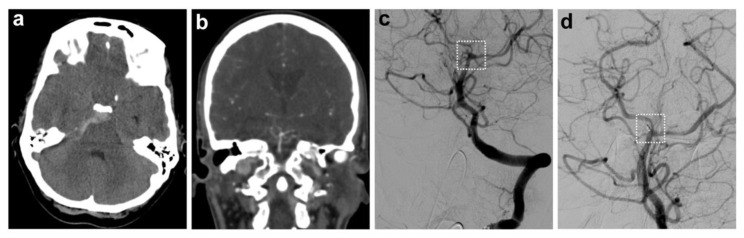
Exemplary case of a patient with a perimesencephalic bleeding pattern and an aneurysmal pathology. (**a**) Non-contrast CT depicting a perimesencephalic pattern of subarachnoid hemorrhage with predominantly right-sided distribution. (**b**) Subsequent CT angiography with evidence of a tiny saccular aneurysm of the SCA on the right, which was initially thought to be a prominent basilar artery. (**c**) DSA confirmed the aneurysm of the right SCA; (**d**) shows the coiled aneurysm (marked with a white dashed box).

Of the 77 patients, 7 (9.1%) were found to have an aneurysm, while 70 (90.9%) had no aneurysmal pathology (exemplary case: Figure 1). In this cohort, initial CTA—performed prior to DSA—identified only 57% of aneurysms (i.e., 4/7 cases). All aneurysms were found in the posterior circulation, originating from small cerebellar arteries, with a maximum diameter of 3 mm.

On admission, the two groups did not differ significantly in their clinical symptoms, including headache (aneurysmal: 100%, non-aneurysmal: 94.3%, *p* = 1.0), neck stiffness (aneurysmal: 14.3%, non-aneurysmal: 27.1%, *p* = 0.67), nausea (aneurysmal: 28.6%, non-aneurysmal: 38.6%, *p* = 0.7), and vomiting (aneurysmal: 0%, non-aneurysmal: 24.3%, *p* = 0.34). Similarly, both groups did not differ with respect to cardiovascular risk factors, including hypertension (aneurysmal: 42.9%, non-aneurysmal: 54.3%, *p* = 0.69), smoking (aneurysmal: 0%, non-aneurysmal: 11.4%, *p* = 1.0), diabetes (aneurysmal: 28.6%, non-aneurysmal: 7.1%, *p* = 0.12), and alcohol consumption (aneurysmal: 0%, non-aneurysmal: 2.9%, *p* = 1.0). Furthermore, mRS at admission did not differ between aneurysmal (1.0 [IQR 1.0–3.0]) and non-aneurysmal patients (1.0 [IQR 1.0–1.0]) (*p* = 0.18), while there were slightly higher WFNS scores in the aneurysmal group (aneurysmal: 1.0 [IQR 1.0–3.5], non-aneurysmal: 1.0 [IQR: 1.0–1.0], *p* = 0.02).

Detailed patient characteristics are shown in Table 1.

### 3.2. Analysis of Complications and Outcome

Complications were more common in patients with aneurysms, although the differences did not reach statistical significance. Hydrocephalus was observed in 42.9% of patients in the aneurysm group compared to 27.1% in the non-aneurysm group (*p* = 0.4). Vasospasm was equally common in both groups, affecting 14.3% of patients in each group (*p* = 1.0). DCI was more common in patients with aneurysms (28.6%) than in those without (7.1%) but did not reach statistical significance (*p* = 0.12).

**Figure 2:**
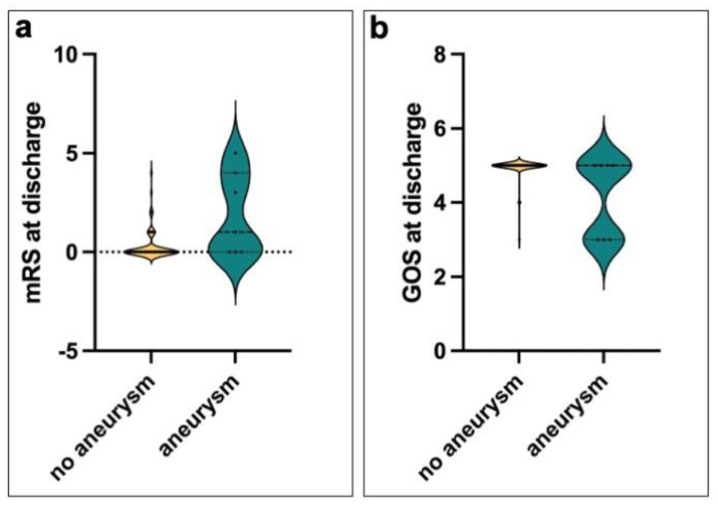
Functional outcome at discharge in patients with and without aneurysmal pathology. The violin plots show the distribution of modified Rankin scale (mRS) scores (**a**) and Glasgow Outcome Scale (GOS) scores (**b**) at hospital discharge, stratified by aneurysm status. While the outcome distribution in patients without aneurysms was tightly clustered around favorable values, the aneurysm group showed a significantly broader distribution with a tendency towards worse outcomes. This pattern reflects greater heterogeneity and more frequent functional impairment in the presence of aneurysmal pathology. Dotted lines indicate medians and shaded areas indicate the density of data points.

Functional outcomes at discharge were significantly worse in patients with aneurysms. The median mRS at discharge was 1.0 [IQR 0.0–3.5] in the aneurysm group compared to 0.0 [IQR 0.0–0.0] in the non-aneurysm group (*p* = 0.0077). The GOS was also lower in the aneurysm group (5.0 [IQR 3.0–5.0]) compared to the non-aneurysm group (5.0 [IQR 5.0–5.0], *p* = 0.0002), reflecting a trend towards worse neurological recovery (Figure 2).

To further investigate predictors of poor functional outcome, a multivariable binary logistic regression was performed, with poor outcome defined as mRS > 2 at discharge as the dependent variable. After adjustment for age, sex, and WFNS score, the presence of an aneurysm emerged as a statistically significant independent predictor, since it was associated with a 21.6-fold increase in the odds of poor outcome (OR = 21.6; 95% CI: 1.00–467.3; *p* = 0.048). None of the other covariates were significantly associated with outcome: age (OR = 0.97, 95% CI: 0.90–1.04, *p* = 0.40), sex (OR = 0.44, 95% CI: 0.06–3.39, *p* = 0.43), and WFNS score (OR = 1.44, 95% CI: 0.82–2.53, *p* = 0.21).

These findings were further supported by ROC analysis. The presence of an aneurysm showed moderate discriminatory power for predicting poor outcome, with an area under the curve (AUC) of 0.72.

## 4. Discussion

This study investigated the impact of the presence of an underlying aneurysmal pathology on the clinical course and functional outcomes of patients presenting with a perimesencephalic bleeding pattern. While aneurysmal cases were rare, their prognostic relevance was significant. Patients with an underlying aneurysm exhibited significantly worse functional outcomes, as reflected by higher mRS and lower GOS scores at discharge. Although complications such as hydrocephalus and DCI occurred more frequently in the aneurysmal group, these differences did not reach statistical significance.

The findings of this study further challenge the common belief that a perimesencephalic bleeding pattern is invariably benign. The current clinical gold standard, DSA, detected all underlying aneurysms, whereas only slightly more than half of the aneurysms (57.1%) were visible on CTA in this study. There are several factors that may account for the limited sensitivity of CTA. These factors include small aneurysm size, aneurysms in anatomically challenging locations (such as near the skull base or in vessels with a tortuous course), and reader experience. This underscores the critical importance of early and sensitive vascular imaging to reliably exclude aneurysms, in line with current international guideline recommendations [14,15,16]. Advanced imaging protocols, such as dual-energy CT or high-resolution CTA, may improve detection rates in future practice.

The clinical presentation of patients with pmSAH is in line with the recent literature, which indicates a frequency of vasospasm of 10–20% (here 14.3%) and a low rate of DCI [17]. While the presence of an aneurysm was significantly associated with poor functional outcome, it was not linked to a higher incidence of complications. This suggests that the less favorable outcome in aneurysmal cases is not primarily mediated by typical secondary complications such as hydrocephalus, vasospasm, or DCI. Hydrocephalus, vasospasm, and DCI are multifactorial and are influenced by blood distribution, inflammatory response, impaired cerebrospinal fluid (CSF) circulation, microvascular dysfunction, and patient-specific factors (e.g., age and anatomy) [18,19,20,21]. Previous studies have shown that vasospasm and DCI can occur regardless of the presence of aneurysms, especially in localized or aneurysm-poor SAH patterns such as pmSAH [22]. The worse outcome in aneurysmal cases may be due to subtle pathophysiological differences, such as different hemorrhage dynamics, vascular reactivity, or microvascular damage, even in the absence of manifest complications [23,24]. The results of this study underscore that functional outcome and complication burden are not the same thing—both need to be considered separately when assessing the prognosis of patients.

Nevertheless, the detection of an aneurysm has significant implications for further clinical management. These include an immediate indication for treatment, such as coiling or clipping to prevent re-bleeding, closer intensive medical monitoring, or adapted anticoagulation [25]. On the other hand, identification of patients without aneurysmal pathology may allow for a less resource-intensive monitoring approach. In confirmed cases of non-aneurysmal pmSAH, early transfer from the intensive care unit (ICU) to a general ward may be appropriate, reducing the length of stay and the burden on the ICU. This distinction is particularly relevant in the current context of increasing patient numbers and limited neurocritical care capacity [26].

Beyond clinical management, the definition of pmSAH remains controversial, particularly in terms of etiological versus anatomic definitions. While classic definitions follow an etiological approach—meaning that the term pmSAH only applies when no aneurysm is found—more recent studies [27] advocate a purely anatomical definition based solely on the radiological bleeding pattern, regardless of the underlying cause. The diagnosis of pmSAH is made when the hemorrhage is confined to the prepontine, interpeduncular, and possibly surrounding cisterns, regardless of whether an aneurysm is present. This anatomical definition has the advantage of being reproducible based on imaging features alone, which facilitates a standardized classification system across centers. However, it disregards clinically relevant etiological distinctions that can significantly affect prognosis and management, especially in cases in which an aneurysm is present despite a perimesencephalic bleeding pattern.

Our results show that the presence of an aneurysm, even with a perimesencephalic bleeding pattern, is associated with a significantly worse functional outcome. This indicates that a definition based on the bleeding distribution alone is clinically inadequate. From a prognostic and therapeutic point of view, it still seems reasonable to define pmSAH etiologically, i.e., to use the term only when a ruptured aneurysm has been excluded. This is the only way to adequately reflect the actual risk constellation of patients and to facilitate treatment stratification.

This study has several limitations. It is a retrospective, single-center analysis, and the aneurysmal group was relatively small (*n* = 7), which limits the statistical power, particularly in subgroup and multivariable analyses. Even if significant differences in functional outcomes could be demonstrated, smaller but clinically relevant effects may remain undetected in this sample. Consistent with this, the small number of aneurysms may have increased the risk of type II errors, especially regarding complications such as hydrocephalus and DCI. Furthermore, due to the small size of the cohort, cross-validation could not be performed to test the generalizability of our predictive model. Future multicenter studies involving larger patient cohorts must address this issue and validate its predictive performance. On the other hand, clinical parameters such as cardiovascular risk factors and symptoms were collected based on existing documentation, meaning that underreporting, e.g., of smoking and alcohol consumption, cannot be ruled out. Another important limitation is the lack of long-term follow-up data (e.g., data collected 3–6 months after discharge). Since outcomes in this study were only assessed at discharge, they may not fully capture patients’ long-term functional recovery or persistent disabilities. Finally, potential confounders such as aneurysm localization, size, or morphology were not included in this analysis but may be prognostically relevant and should be considered in future studies.

## 5. Conclusions

This study demonstrates that in patients with a perimesencephalic hemorrhage, the presence of an underlying aneurysm is associated with significantly worse functional outcomes. These findings underscore the prognostic relevance of aneurysm detection and support the continued need for early, sensitive vascular imaging—particularly DSA—to reliably exclude aneurysmal pathology. From both a clinical and organizational perspective, the distinction between aneurysmal and non-aneurysmal pmSAH remains essential for target management and resource allocation.

## Figures and Tables

**Table 1:** Comparison of baseline characteristics, complications, and outcomes between patients with and without aneurysmal pathology. The only continuous variable, age, was normally distributed in both groups according to the Shapiro–Wilk test and is therefore presented as the mean ± SD and was compared using an unpaired *t*-test. All ordinal variables are presented as the median and IQR and were analyzed using the Mann–Whitney U test. Categorical variables are presented as counts and percentages and were compared using Fisher’s exact test or the chi-squared test, depending on group size. DCI: delayed cerebral ischemia, GOS: Glasgow Outcome Scale, IQR: interquartile range, mRS: modified Ranking scale, SD: standard deviation, WFNS: World Federation of Neurosurgical Societies.

**Parameter**	**Non-Aneurysmal** **(*n* = 70)**	**Aneurysmal** **(*n* = 7)**	***p*** **-Value**
Age *(years, mean ± SD)*	55.1 ± 11.7	55.9 ± 13.8	0.897
Sex *(n (%))*			
Male	44 (62.9%)	2 (28.6%)	0.11
Female	26 (37.1%)	5 (71.4%)	0.24
WFNS score at admission *(median [IQR])*	1.0 [1.0–1.0]	1.0 [1.0–3.5]	0.02
mRS at admission *(median [IQR])*	1.0 [1.0–1.0]	1.0 [1.0–3.0]	0.18
Clinical symptoms *(n (%))*			
Headache	66 (94.3%)	7 (100%)	1.0
Neck Stiffness	19 (27.1%)	1 (14.3%)	0.67
Nausea	27 (38.6%)	2 (28.6%)	0.7
Vomiting	17 (24.3%)	0 (0%)	0.34
Cardiovascular risk factors *(n (%))*			
Hypertension	38 (54.3%)	3 (42.9%)	0.69
Smoking	8 (11.4%)	0 (0%)	1.0
Diabetes	5 (7.1%)	2 (28.6%)	0.12
Alcohol	2 (2.9%)	0 (0%)	1.0
Complications *(n (%))*			
Hydrocephalus	19 (27.1%)	3 (42.9%)	0.4
Vasospasm	10 (14.3%)	1 (14.3%)	1.0
DCI	5 (7.1%)	2 (28.6%)	0.12
Clinical outcome *(median [IQR])*			
GOS at discharge	5.0 [5.0–5.0]	5.0 [3.0–5.0]	0.0002
mRS at discharge	0.0 [0.0–0.0]	1.0 [0.0–3.5]	0.0077

## Data Availability

The original contributions presented in this study are included in the article. Further inquiries can be directed to the corresponding authors.
